# Metastatic canine mammary carcinomas can be identified by a gene expression profile that partly overlaps with human breast cancer profiles

**DOI:** 10.1186/1471-2407-10-618

**Published:** 2010-11-09

**Authors:** Robert Klopfleisch, Dido Lenze, Michael Hummel, Achim D Gruber

**Affiliations:** 1 Department of Veterinary Pathology, Freie Universität Berlin, Robert-von-Ostertag-Straße 15, 14163 Berlin, Germany; 2 Charité Universitätsmedizin Berlin, Campus Benjamin Franklin, Institute of Pathology, Hindenburgdamm 30, D-12200 Berlin, Germany

## Abstract

**Background:**

Similar to human breast cancer mammary tumors of the female dog are commonly associated with a fatal outcome due to the development of distant metastases. However, the molecular defects leading to metastasis are largely unknown and the value of canine mammary carcinoma as a model for human breast cancer is unclear. In this study, we analyzed the gene expression signatures associated with mammary tumor metastasis and asked for parallels with the human equivalent.

**Methods:**

Messenger RNA expression profiles of twenty-seven lymph node metastasis positive or negative canine mammary carcinomas were established by microarray analysis. Differentially expressed genes were functionally characterized and associated with molecular pathways. The findings were also correlated with published data on human breast cancer.

**Results:**

Metastatic canine mammary carcinomas had 1,011 significantly differentially expressed genes when compared to non-metastatic carcinomas. Metastatic carcinomas had a significant up-regulation of genes associated with cell cycle regulation, matrix modulation, protein folding and proteasomal degradation whereas cell differentiation genes, growth factor pathway genes and regulators of actin organization were significantly down-regulated. Interestingly, 265 of the 1,011 differentially expressed canine genes are also related to human breast cancer and, vice versa, parts of a human prognostic gene signature were identified in the expression profiles of the metastatic canine tumors.

**Conclusions:**

Metastatic canine mammary carcinomas can be discriminated from non-metastatic carcinomas by their gene expression profiles. More than one third of the differentially expressed genes are also described of relevance for human breast cancer. Many of the differentially expressed genes are linked to functions and pathways which appear to be relevant for the induction and maintenance of metastatic progression and may represent new therapeutic targets. Furthermore, dogs are in some aspects suitable as a translational model for human breast tumors in order to identify prognostic molecular signatures and potential therapeutic targets.

## Background

Canine mammary tumor (CMT) is the most common cancer among female dogs and often becomes fatal due to the development of distant metastases [1-3]. Metastasis to the regional lymph node is an early step in metastasis and one of the most important prognostic factors in the diagnosis of CMT, a criterion that is also valid for human breast cancer [4,5]. Lymph node metastases of CMT are usually followed by the development of distant metastases, mainly in the lung, ultimately leading to the death of the dog [6]. However, knowledge of the molecular mechanisms contributing to lymph node and distant metastasis is still fragmentary. Despite numerous studies on this issue, significant metastasis-associated and predictable expression patterns of single genes have not been identified in CMT as yet [7-9]. Global gene expression profiles that compare metastasizing versus non-metastasizing CMT are unavailable whereas several studies on human breast cancer found significant metastasis associated expression profiles. The latter studies identified several non-overlapping expression signatures which are related to the development of lymph node and distant metastases and worse prognoses [10-13]. The available studies on global gene expression in CMT compared normal mammary gland, benign and malignant tumors with unknown lymph node status and clinical follow-up [14,15]. The authors reported that the gene expression profiles of CMT include a gene expression signature associated with neoplastic transformation. Furthermore, comparison of canine and human expression profiles disclosed an overlap of deregulated genes in human and canine mammary tumors [15]. Generally, clinical and molecular features of human and canine bear a likeness in several aspects. Both malignancies are the most common cancer of the female, lymph node metastases indicate a poor prognosis, the hormonal status influences the development of CMT and estrogen receptor (ER), progesterone receptor (PR) and ERBB2 expression patterns do influence the overall survival rate [1,16-18].

The aim of this study was the identification of gene expression signatures in primary CMT that are associated with early lymph node metastasis. Global mRNA expression profiles obtained from metastatic versus non-metastatic CMT cases were compared and differentially expressed genes were analyzed for their function and their role in pathway activation. Moreover, mining in published literature revealed interesting overlapping features when compared to data derived from gene expression profiles of metastatic human breast carcinomas.

## Methods

### Tissue samples

Thirteen simple mammary carcinomas with invasive growth and lymph node metastases at the time of tumor resection and 14 simple carcinomas without lymph node metastases were included in the study (Table 1). Complex carcinomas were excluded from the study to avoid differences in gene expression levels due to differences in mesenchymal/epithelial ratio in the different tissues. None of the patients had a history of progestin treatment or radiographically detectable pulmonary metastases at the time of tumor resection. Distant metastases as the cause of death were determined postoperatively by radiographic detection of metastases (nos. 1-11) or necropsy (nos. 12 and 13). Selection criteria for carcinomas without lymph node metastases included an invasive growth, a negative lymph node status, a histological grade III and a minimal tumor diameter above the average of the lymph node positive tumors (> 2.42 cm). All animals with non-metastatic carcinomas had a overall survival rate of over 24 months except animal no. 22 which developed radiographic detectable lung metastases 8 months after surgery.

**Table 1:** Tumors, lymph node status and histologic and immunohistochemical features of metastatic and non-metastatic canine mammary carcinomas

Dog	Breed	Age in years	Histologic Grade	Tumor size (cm)	Affected lymph node	Postoperative overall survival (months)	ERa	ERBB2
1	American Pittbull	9	Grade III	3	Inguinal	8	-	+
2	Bobtail	10	Grade III	3	inguinal	6	-	+
3	Rottweiler	10	Grade III	3	inguinal	6	-	+
4	Dachshund	13	Grade II	2	inguinal	3	-	+
5	Bavarian Mountain Dog	13	Grade III	5	inguinal	8	-	+
6	Golden Retriever	11	Grade III	1	inguinal	5	-	-
7		12	Grade II	1	axillary	2	-	+
8	Pudel	16	Grade II	3	inguinal	2	-	-
9	West Highland White Terrier	16	Grade II	0.5	inguinal	3	-	+
10	French Bulldog	15	Grade III	1	inguinal	/	-	+
11	Mixed Breed	16	Grade II	3	inguinal	9	-	+
12	Beagle	11	Grade II	4	inguinal	1	-	+
13	Mixed Breed	9	Grade III	2	inguinal	/	-	+

14	Mixed Breed	12	Grade III	4	-	>24	-	+
15	Dalmatian	10	Grade II	3	-	>24	-	+
16	Irish Setter	9	Grade II	3	-	>24	-	+
17	Labrador	11	Grade III	3	-	>24	-	-
18	Soft coat. wheaten Terrier	12	Grade III	4	-	>24	-	+
19	Mixed Breed	11	Grade III	3	-	>24	-	+
20	German Short Hair	12	Grade III	15	-	>24	-	+
21	Poodle	11	Grade II	3	-	>24	-	+
22	Mixed Breed	15	Grade III	7	-	>8	-	+
23	Miniature Pinscher	13	Grade II	2	-	>24	-	+
24	Mixed Breed	11	Grade III	9	-	>24	-	+
25	Great Dane	10	Grade III	7	-	>24	-	+
26	Mixed Breed	9	Grade III	3	-	>24	-	+
27	Mixed Breed	13	Grade III	7	-	>24	-	+

WHWT: West Highland White Terrier

The experimental research reported in the manuscript has been performed with the approval of animal welfare authorities and the ethical committee of the Freie Universität Berlin. Surgical excision of tumour biopsies was part of the tumour treatment according to the state of the art treatment and solely to improve the animals welfare. Furthermore, the animals were under full anaesthesia and not exposed to any additional manipulation due to the inclusion in this study. All animal owners received and approved an informed client consent form.

Tissue specimens were fixed in neutral-buffered 4% formalin or snap frozen in liquid nitrogen within 15 minutes after resection and stored at -80°C until further use. Formalin fixed tumor tissue samples were routinely embedded in paraffin and sections of 2-μm were stained with hematoxylin and eosin. Tumor and lymph node histologies were evaluated independently by two board-certified pathologists, following the criteria of the WHO classification of canine mammary tumors and the Nottingham grading system [19,20]. All 27 tumors were simple carcinomas and characterized by an invasive, mostly solid growth pattern, marked cellular pleomorphism, anisokaryosis and 3 or more mitotic figures per high power field.

### Immunohistochemistry

Oestrogen receptor alpha (ER), ERBB2 expression was immunohistochemically determined using the ABC-method. In brief, monoclonal mouse anti-human ER specific antibody (1:1000, clone CC4-5, Novocastra, Wetzlar, Germany) and rabbit polyclonal anti-human ERBB2 specific antibody (1:150, cat. no. A0485, Dako, Hamburg, Germany) were diluted in Tris-buffered saline (TBS, 50 mM, pH 7.6) and incubated at 4°C overnight after a blocking step with 50% goat serum in TBS for 30 min at room temperature. Polyclonal goat anti-rabbit IgG (1:200; Vector, England, BA1000) and goat anti-mouse IgG (1:200; BA9200, Vector, Burlingame, USA) were used as secondary antibody. Diaminobenzidine tetrahydrochloride (D8001, Sigma Aldrich, Munich, Germany) was used as chromogen and slides were counterstained with hematoxylin (Merck). Normal canine and human mammary gland were used as positive tissue controls. ER and ERBB2 immunolabeling was evaluated in 10 random 200× magnification fields. Tumors were defined as ER or ERBB2 positive if more than 10% of the cells stained positive with the respective antibody.

### Macrodissection of tumor samples and RNA isolation

Macrodissection was performed on all tumor specimens to ensure high tumor cellularity. Only sections with more than 70% carcinoma cells were included in the study as shown by digital image analysis (Scanscope T3, Aperio, Vista, USA; Zeiss Axiovision, Jena, Germany). For mRNA isolation, tissue sections were transferred into 300 μl of lysis buffer (NucleoSpin RNA; Macherey&Nagel, Dueren, Germany) and homogenized (Precellyse 24, Bertin Technology, France). RNA was extracted and purified using a commercial system (NucleoSpin RNA; Macherey&Nagel, Düren, Germany). The RNA quality was controlled using the BioAnalyzer (Agilent Technologies, USA) and only high quality RNA (RIN >8) was used for microarray analyses. For reverse transcription a commercial kit (Iscript cDNA synthesis Kit, Biorad, Germany) was used. 100 ng of total RNA of each sample were used for reverse transcription of each tissue sample and the reactions were performed exactly according to the manufacturers instructions. cDNA was stored in low adhesion tubes at -20°C.

### Microarray analyses

Affymetrix GeneChip hybridization (Canine Genome 2.0 Array) was performed with 2 μg total RNA according to the manufacturer's recommendations. The chips were stained and washed with the GeneChip Fluidics Station 450 and visualized on an Affymetrix GeneChip Scanner 3000. Microarray data were deposited at the Gene Expression Omnibus data repository under the number GSE20718: (http://www.ncbi.nlm.nih.gov/geo/query/acc.cgi?token=bbwvhacsemokkfq&acc=GSE20718)

### Quantitative RT-PCR (qPCR)

Quantitative PCR was used to validate array gene expression data. To this end, established qPCR assays for eight genes that were identified in the metastatic gene signature were used to compare mRNA expression levels in metastatic and non-metastatic tumors. Primer sequences, product length and sequence accession numbers for the PCR assays for AURKA, ALOX12, BMP6, ERBB4, HEPACAM2, IGFR2, RAD51, TGFBR3 and the housekeeper genes A5B, HPRT and RP32 mRNA expression levels were used before [17,21-24] and are describe in Additional file 1. Each qPCR reaction had a 20 μl reaction volume containing 5 μl cDNA corresponding to 100 ng input RNA, 500 nM of each forward and reverse primer, 12.5 μl of the Maxima Sybr Green/Rox qPCR Master Mix (Fermentas, Germany) containing 2.5 mM Mg^2+ ^and 5 mM dNTP. Tubes and lids were purchased from Fermentas (Germany). Cycling parameters for all assays were, 95°C for 10 min, 95°C for 30 sec, 58°C for 60 sec, 72°C for 30 sec for 40 cycles. Melting curve ranged from 58°C to 95°C and was read every 0.2°C and hold for 2 sec. The cDNA of all samples were amplified on the same plate for every primer pair to ensure equal amplification conditions. Specificity of amplification products was confirmed by melting curve and sequence analyses. For each sample, results were documented as cycle threshold (CT) set to 100 relative fluorescence unit values of background subtracted qPCR fluorescence kinetics by using the MX Pro Stratagene analysis software, applying the adaptive baseline, amplification based threshold and moving average algorithm enhancement. Relative expression of the target gene (TG) was calculated using a comparative ΔCT-method with multiple housekeepers as previously described[25-27]. The housekeeper genes used were selected from a panel of reference genes (RG) according to the GeNorm algorithm[28]. Data are presented as fold change in gene expression level of the gene of interest (GOI) in lymph node-positive carcinomas (LN+) normalized to the housekeepers and to the similarly normalized GOI expression levels in lymph node-negative carcinomas (LN-). Significance (p < 0.05) of differential gene expression was tested using Shapiro-Wilk-test and t-test.

### Data analysis

Affymetrix CEL files were imported into Partek Genomic Suite Software (Version 6.4, Partek Inc., St. Louis, USA) and processed by the implemented gcRMA workflow (median polish probe set summarization, RMA background correction, quantile normalization). Differences in gene expression between samples with and without metastases were analyzed by ANOVA and false discovery rate was controlled by using the q-value method. Differentially expressed genes were selected by applying a filter of q < 0.001 and a fold-change of >1.7 in both directions. Unnamed genes were excluded from the list. Hierarchical clustering of the samples and genes was conducted using Pearson correlation and complete linkage.

To supplement the gene annotations of the differentially expressed genes with functional information, BLAST search and Affymetrix-provided human to canine microarray comparisons were used to map canine genes to their human equivalents as has been shown before [29]. Using the human equivalents as templates, the DAVID database was queried for gene ontology information [30]. To study enriched functional gene families and functional annotation, all down-regulated and all up-regulated genes were submitted separately to DAVID. In the case of redundant probe with a fold change in the same direction only the probe set with the highest fold change was included in further analysis. Selection criteria for DAVID included a medium stringency, ≥ 4 probes within a cluster and an enrichment factor >1.3. In case of multiple appearances of similar gene families or functional annotation terms, the cluster with the higher enrichment factor was selected. Reported associations between the differentially expressed genes and human breast cancer were determined using the MEDGENE database [31], entering the search term: 'breast neoplasms'.

The GenMAPP software was used for pathway analysis [32]. Analyses of up- or down-regulated gene sets were performed independently. Quality criteria for pathway selection included more than 3 genes, z-score of |>2| and p < 0.05.

## Results

### Immunohistological phenotype of the tumors

All lymph node positive and lymph node negative tumors were ER negative. In addition, 11/13 lymph node positive tumors and 13/14 lymph node negative tumors were ERBB2 positive.

### Identification of differentially expressed genes

**Figure 1:**
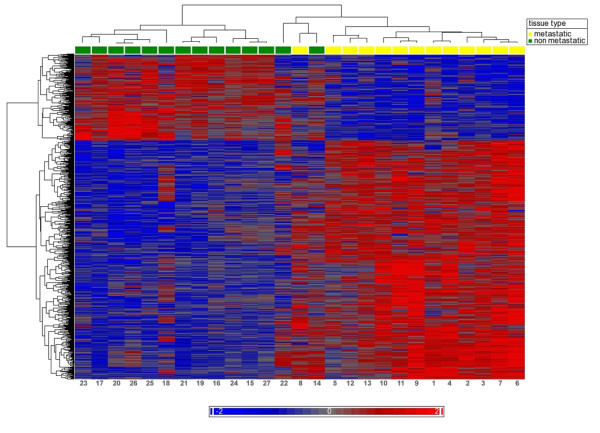
**Two dimensional cluster analysis of metastatic versus non-metastatic canine mammary tumors**. Two of the lymph node metastasis-negative carcinomas were adjoined to the metastatic tumor cluster. One of these animals (no. 22) developed lung metastases 8 months after initial surgery despite negative lymph-node status.

Comparison of the global expression of canine mammary carcinomas with or without lymph node metastasis revealed 1011 differentially expressed genes, 744 genes of which were up-regulated metastatic carcinomas whereas only 267 genes were down-regulated (1.7× fold change, false discovery rate q < 0.001, Additional file 2). With the exception of two cases (dogs no. 14, 22), these genes were able to clearly separate the two tumor groups by means of two-dimensional hierarchical clustering (Figure 1). Interestingly, one of the tumors (no. 22) developed radiographic detectable lung metastases 8 months postoperatively. However, the other case (14) did not develop metastases during a 24 month clinical follow-up. qPCR confirmed significant expression differences for all seven of the eight genes analyzed (Table 2).

**Table 2:** Confirmation of cDNA-Array data by quantitative RT-PCT

Gene	Mean fold change in metastatic carcinomas by qPCR	Mean fold change in metastatic carcinomas by microarray
AURKA	2.5 (0.52)*	5.9
ALOX12	0.6 (0.11)*	0.4
BMP-6	2.4 (0.97)*	4.4
ERBB-4	0.4 (0.09)*	0.4
HEPACAM2	0.2 (0.05)*	0.01
IGFR2	0.6 (0.11)	0.4
RAD51	2.4 (0.67)*	4.7
TGFBR-3	0.41 (0.90)*	0.2

* Difference between mean expression levels significant (p < 0.05)

### Up-regulation of cell cycle and DNA-damage repair genes in metastatic canine carcinomas

Functional analysis of the metastasis-associated genes identified an up-regulation of 93 cell cycle associated genes with functions in cell cycle progression (Table 3). Interestingly, the 13 cell cycle checkpoint genes and 27 DNA-damage repair genes in the gene list were exclusively up-regulated and only one of the 6 cyclin genes was down-regulated (Table 3). 49 genes associated with protein metabolism were also up-regulated, including protein folding, ubiquitin dependent proteins and prefoldins, suggesting an increased biosynthesis of the cells. Furthermore, anti-apoptotic and pro-apoptotic genes, members of the RAS signaling cascade were up-regulated (Table 4). Interestingly, transcription factors and methyltransferases were significantly enriched in both up-regulated and down-regulated gene lists indicating that the metastatic phenotype is associated with a different equilibrium of gene activation and deactivation (Table 4).

**Table 3:** Up- and down-regulated cell cycle genes

Up-regulated cell cycle genes	Down-regulated cell cycle genes
Cell Cycle Genes	

ANAPC10, APPL1, AURKA, BIRC5, BUB3, CCDC5, CCNA2, CCNB1, CCNB2, CCNE1, CDC2, CDC27, CDC37, CDC45L, CDC5L, CDC6, CDCA2, CDCA5, CDCA8, CETN3, CGREF1, CHAF1A, CHAF1B, CHEK2, CKAP2, DKC1, DTYMK, ERBB2IP, ESCO2, ESPL1, EXO1, FBXO5, GADD45B, GADD45GIP1, GMNN, GTSE1, ILF3, INCENP, IRF1, KATNA1, KIF15, KIF23, KIFC1, LIN9, MAD2L1, MCM5, MCM6, MIS12, MRPL41, MYBL2, NCAPG, NCAPG2, NCAPH, NDC80, NME1, NOLC1, NPM1, NUF2, NUSAP1, PARD3B, PBK, PES1, PFDN1, PIK3CB, PLK1, PPM1G, PRC1, PRMT5, PTPN11, RAD17, RAD21, RAD51, RANBP1, RASSF1, RASSF4, B1CC1, RBL1, RPA1, SASS6, SGOL1, SMC1A, SPC24, SPC25, SPIN1, STK11, SUGT1, TPX2, TTK, TXNL4A, UHRF1, YEATS4, YWHAQ, ZW10	BCL6, DBC1, DST, KHDRBS1, LATS1, NEDD9, PPP2R1B, RBM5, SESN1, TARDBP,
Cell Cycle Checkpoint Genes	

BIRC5, BUB3, CDC2, CDC45L, CDC6, CHEK2, GTSE1, MAD2L1, PTPN11, RAD17, SMC1A, TTK, ZW10,	
Cyclin Genes	

CCNE1, RBL1, CCNB2, CCNA2, CCNB1	CCNL2
DNA-Damage Repair Genes	

APEX2, CHAF1A, CHAF1B, DCLRE1B, ESCO2, EXO1, FEN1, GADD45B, GADD45GIP1, GEN1, GTF2H3, NSMCE2, NUDT1, POLD3, RAD17, RAD21, RAD51, RAD51C, RFC3, RPA1, SMC1A, SMC5, TOPBP1, TYMS, UBE2N, UHRF1, UNG	

**Table 4:** Enriched functional gene classes in the differential expression profile of metastatic canine mammary tumors

Functional gene class	Gene number	Enrichment factor
**up-regulated genes**		
M-phase	52	17.49
DNA replication	29	6.72
Protein folding	32	6.34
Transcription factors	48	6.21
RNA processing	50	5.99
Apoptosis	10	5.98
Mitochondrion	59	5.96
Cell cycle checkpoint	14	4.09
DNA damage response	30	3.52
Ubiquitin-pathway	13	2.56
DNA-replication initiation	7	2.05
Methyltransferases	15	1.95
RAS-signalling	5	1.71
Prefoldins	4	1.62
Transcription repressors	13	1.32

**down-regulated genes**		
Transcription factors	55	3.62
mRNA processing	11	3.45
Muscle contraction	10	2.86
Transcription regulation	41	2.36
Cell growth regulation	10	2.03
Methyltransferases	6	1.75
Cell differentiation	54	1.58
Transmembrane tyrosine kinase receptors	12	1.33

### Down-regulation of growth factor receptor pathways and cell differentiation genes in metastatic canine carcinomas

39 genes with receptor activity were up- and down regulated in metastatic tumors (Table 4, 5). However, transmembrane tyrosine kinase receptor genes were significantly and exclusively down-regulated in metastatic carcinomas (Table 5). Specifically, genes of growth factor signaling pathways epidermal growth factor (*EGF*), transforming growth factor-beta (*TGFB*), fibroblast growth factor (*FGF*), hepatocyte growth factor receptor (*MET*) and insulin-like growth factor (*IGF*) I and II were preferentially down-regulated in the metastatic tumors. Besides their growth stimulatory effects, *EGF*, *TGF *and *FGF *signaling also induces cell differentiation in mammary epithelial cells and a down regulation of these pathways and associated proteins has been described before [24,33-37].

**Table 5:** Up- and down-regulated receptors genes

Up-regulated receptor genes	Down-regulated receptor genes
Receptors	
CD300LF, CRLF3, F2RL1, FKBP3, GOSR2, HYAL2, IL10RB, ITGA4, JMJD6, KIF15, LBR, MED30, PFDN6, RNF139, SIVA1, TFRC, TREM1, ZNHIT3	EPHB1, EPHB3, ERBB4, FGFR1, FKBP, GIT2, GPR116, IGF2R, MET, NISCH, NRP1, NPR2, NRXN1 NTRK2, PRKCZ, PTPN2, TGFBR3, THRB, TNFRSF1B, TNFRSF21, TNPO3
Transmembrane Receptors with Tyrosine Kinase Activity	

	EPHB1, EPHB3, ERBB4, FGFR1, IGF2R, MET, NRP1, NTRK2, TGFBR3,
G-Protein Coupled Receptors	

F2RL1, SLC26A6	GPR116, NPR2
Cell Differentiation Genes	

AATF, ASF1B, Bak1, BCLAF1, BID, BIRC3, BIRC5, BMP6, BNIP2, CASC5, CASP6, CDC2, CEP57, CHEK2, CKAP2, CLN5, CNP, CSE1L, DNAJA3, DNM1L, EIF2B2, ESPL1, FASTKD3, FBXO5, GADD45B, HRB, HSPD1, JMJD6, KATNA1, MRPL41, MYBL2, NME1, NPM1, OPA1, PDCD10, PEX13, PLDN, PRKAA1, PTPN11, RAD21, RB1CC1, RNF6, RNF7, SAP30BP, SFXN1, SIVA1, SKIL, SLTM, SON, STK3, STK4, STRBP, TGM2, WHSC1L1, YWHAG	ACTN1, AFF4, ALOX12, BCL6, CATSPER1, CSPG5, DBC1, EPHB3, ERBB4, FGFR1, FST, GATA3, MET, MYH11, NRP1, NRXN1, NTF3, NTRK2, PIK3R1, PPP2R1B, PRKCZ, SEMA6D, SMARCA1, SOX5, SOX6, TBX19, THRB, TNFRSF1B, TNFRSF21, TSG101, ZBTB16

In line with this changed receptor signaling in metastatic carcinomas is therefore the de-regulation of several genes associated with cell growth regulation and cell differentiation in the metastatic tumors (Table 5). Interestingly, statistical analysis by DAVID gene ontology identified a significant enrichment of cell differentiation genes only in the list of down-regulated genes (Tables 4).

### Cell-cell adhesion and matrix modulation genes are differentially expressed in metastatic tumors

It is commonly accepted that metastasis requires loss of cell-cell adhesion and expression of extracellular matrix modulators by the tumor cells. We, therefore, specifically screened the gene list for modulators of the extracellular matrix, cell adhesion and angiogenesis genes. Statistical analysis found no significant enrichment of matrix modulator genes in the gene list (Table 4). However, three matrix modulator genes: matrix-metalloproteinase 11, cathepsin L2 and the pro-cancerogenic serin peptidase inhibitor E1, were up-regulated in metastatic tumors (Table 6) and this is in agreement with earlier observations in human breast cancer [38-40]. Similarly, cell adhesion genes were also not significantly enriched in the gene list. However, the 20 adhesion and focal adhesion complex genes present in the differential gene list were predominantly down-regulated, like *HEPACAM2, EMILIN1, PCDHGC3 *and *ASAM *but some adhesion genes were also up-regulated, like *ICAM2 *and the *ITGA4 *(Table 6). A similar expression pattern for several of these genes has been described for human breast cancer and other cancer types before (Table 6) [23,41-44].

**Table 6:** Up- and down-regulated matrix modulator, cell adhesion and focal adhesion complex genes

Up-regulated genes	Down-regulated genes
Matrix Modulators	

MMP11, SERPINE1, CTSL2	
Cell adhesion	

DST, ICAM2, ITGA4, ITGB1BP1, LBR	ACTN1, ALOX12, ASAM, BCL6, CHAD, COL16A1, CYR61, DST, EMILIN1, HEPACAM2, MIA3, NEDD9, NRP1, NRXN1, PCDHGC3
Focal Adhesion	

PTK2	ACTN1, BIRC3, CHAD, COL2A1, MET, MYLK, PIK3R1

Finally, analysis of the gene list disclosed no significant enrichment of angiogenesis genes. The 7 angiogenesis genes present in the list were also predominantly down-regulated (e.g. *NRP1, JMJD6, CYR61, FGFR1*) and again the impact of these genes on the carcinogenesis of breast cancer cells has been described[36,45,46].

### Comparison with human breast cancer expression profiles

The identified metastasis-associated expression profile of canine mammary tumors has several significant overlaps with expression profiles of metastatic human breast cancer. Literature mining by Medgene disclosed that 25% of the canine gene list has been cited in association with human breast cancer before. However, the mining approach by Medgene is based on information presented in the abstract of the articles and not complete gene expression profiles [31]. It is therefore likely that a comparative meta-analysis of gene expression profiles will identify additional overlapping gene expression patterns. The overlapping genes are mostly associated with cell division, cell growth, kinase activity, translation, DNA integrity checkpoint and transcription regulation.

GenMapp pathway analysis identified a marked overlap of deregulated pathways in the canine metastatic profile and a recent meta-analysis on metastasis-associated gene expression profiles (Table 7) [10]. All 5 significantly up-regulated pathways were Identical to human breast cancer the cell cycle, G1 to S control reactome and DNA replication reactome pathways were up-regulated, whereas striated muscle contraction and IL-6 pathways were significantly down-regulated. (Table 7).

**Table 7:** Up- and downregulated pathways in metastatic carcinomas when and (GenMAPP analysis)

Dysregulated pathways in metastatic canine mammary tumors	Gene number	Dysregulated pathways in metastatic human breast cancer
**up-regulated pathways**		Ranking in a meta-analysis[1]
Cell_cycle	33	1
Cell_cycle_G1_to_S_control_reactome	22	2
DNA_replication_reactome	25	3
Tissue_embryonic_stem_cell	11	6
Proteasome degradation	7	7

**down-regulated pathways**		
Striated_muscle_contraction	8	4
Focal_adhesion	11	/
IL-6_NetPath_18	5	6

/not present in the metastatic pathways analysis of human breast cancer

Furthermore, comparison with the van't Veer 70 gene prognostic signature which reliably identifies human breast cancers with metastatic potential also found a significant overlap [13]. However, only 34 of the 70 genes are present on the canine gene array but 23 (68%) of these genes were included in the metastatic gene expression profile of CMT indicating that comparable prognostic gene expression patterns may exist in canine mammary tumors (Table 8).

**Table 8:** Identification of canine genes in the expression profile of metastatic CMT homologous or similar to the 70-gene prognostic signature for human breast cancer

Human genes of the 70 prognostic gene profile (van't Veer et al.)	Homologous genes expressed in metastatic canine tumors	Gene family members expressed in canine tumors
Homologous genes present on canine gene array		
AP2B1	AP2B1	-
CCNE2	-	CCNE1
CDC42BPA	-	-
CENPA	CENPA	-
COL4A2		COL2, 16
DCK	DCK	-
DIAPH3	DIAPH3	-
ECT2	ECT2	-
EGLN1	-	-
ESM1	ESM1	-
EXT1	-	-
FGF18	-	-
FLT1	-	-
GMPS	-	GMPS
GNAZ	-	-
HRASLS	-	-
IGFBP5	-	-
LPCAT1	LPCAT1	-
MCM6	MCM6	-
MELK	MELK	-
MMP9	-	MMP11
MS4A7	-	-
NDC80	NDC80	-
ORC6L	ORC6L	-
OXCT	-	-
PECI	-	-
PRC1	PRC1	
RAB6B	-	RAB10, 7L1, EPK
RFC4	-	RFC2, 3
SCUBE2	-	-
SLC2A3	-	-
STK32	-	STK3, 4, 11
TGFB3	-	-
UCHL5	-	UCHL3
ALDH4	-	ALDH16
FAM2A	-	FAM13B
NMU	-	NMB

## Discussion

In the present study we identified a gene expression profile in CMT that is associated with early metastatic spread to the lymph nodes and is able to discriminate carcinomas with similar histological features but divergent metastatic potential. The differential expression profile contains several enriched functional gene classes and has significant overlaps with expression profiles of metastatic human breast cancer.

The similarities found between canine and human mammary tumors in terms of increased proliferation, altered cell differentiation status and decreased cell adhesion were also confirmed by Medgene literature mining. Approximately 25% of the deregulated genes in metastatic canine carcinomas have been cited in association with human breast cancer. In this subset of cited genes, a significant enrichment of genes associated with cell cycle regulation, protein kinases, DNA integrity checkpoint and protein metabolism was observed. In addition, more than 60 percent of the van't Veer 70-gene prognostic signature was identified in our differential gene list. It is therefore likely that gene expression profiles may also predict metastasis in CMT. For instance, one of the dogs without lymph node metastases at the time of investigation developed later distant metastases. Strikingly, this case clustered within the group of metastatic tumors indicating that metastatic potential can be identified in CMT before clinically and morphologically overt metastases. It will be therefore of interest to compare the gene expression profiles of lymph node negative CMT with a comprehensive clinical follow-up to identify potential predictive patterns similar to human breast cancer.

Generally, metastatic CMT were characterized by increased expression of cell division genes. These findings are identical to a recent meta-analysis on expression profiles of metastasizing human breast cancer which found cell cycle and DNA replication pathways most significantly up-regulated [10,13,47]. Unexpectedly, this expression profile of increased proliferation was accompanied by significantly decreased growth factor receptor expression. Increased proliferation and malignant transformation of most cancer types is at least in part commonly attributed to aberrant growth factor signaling. In addition, growth factor signaling is also a major differentiation impulse. The decreased growth factor pathway expression in metastatic carcinomas hence may be a possible cause for the altered expression of cell differentiation and unlocked cell growth regulation genes in these CMT. The dysregulation of cell cycle control is therefore a dominant, cross species feature metastatic mammary tumors. Common "driver" mutations of this cell proliferation in the tumor genome of both species are unknown at the moment. The partly overlapping transcriptome of metastatic canine and human carcinomas, however, indicate similar mechanisms of mammary carcinogenesis and a suitable molecular model character of canine mammary tumors.

Genes associated with focal adhesion and regulation of actin cytoskeleton organization were significantly down-regulated in metastasizing CMT. This is in agreement with the observation that loss of cell to cell contact is a major step in the metastatic process and has been observed in canine mammary tumors before [48,49]. The effect of the down-regulated pathway of actin regulation is somewhat unclear, but disorganization of the actin cytoskeleton is a common feature of malignant breast cancer cells [50]. Of interest qPCR analyses confirmed the differences in mRNA expression levels in metastatic and non-metastatic carcinomas although the fold change in expression difference consistently lower in the qPCR assays when compared with microarray assays for all eight genes analyzed. The eight genes, *AURKA, ALOX12, BMP6, ERBB4, HEPACAM2, IGFR2, RAD51, TGFBR3*, were selected due to their description in the literature on canine mammary tumors before[17,21-24,51]. Their gene expression has been described metastatic carcinomas, metastases, adenomas and normal mammary gland and these studies showed a similar tendency of changes in gene expression between benign and malignant canine mammary tissue types[17,21-24,51]. Of note, a comparison of the proteome of the same subset of tumours did not identify similar proteins[52].

## Conclusion

In conclusion, our findings show that metastatic spread of CMT to the lymph nodes is associated with a gene expression profile of increased cell cycle progression, altered cell differentiation and decreased growth factor signaling. Furthermore, several matrix modulators and cell adhesion genes have an altered expression in metastatic tumor cells while angiogenesis gene expression is not differentially regulated in metastatic and non-metastatic CMT. Several major aspects of metastasis-associated gene expression are therefore similar between human and canine mammary tumors. Furthermore, the differential expression profiles in metastatic and non-metastatic carcinomas allow a prediction of clinical behavior and offer several new hypotheses for molecular pathways and networks involved in the metastatic process of canine mammary tumors.

## List of abbreviations

ASAM: adipocyte-specific adhesion molecule; BLAST: basic local alignment search tool; CMT: canine mammary tumour; CYR61: cysteine-rich, angiogenic inducer 61; DAVID: database for annotation, visualization and integrated discovery; EGF: epidermal growth factor; EMILIN1: elastin microfibril interfacer 1; ER: estrogen receptor; ERBB2: epidermal growth factor receptor 2; FGF: fibroblast growth factor; FGFR1: fibroblast growth factor receptor 1; HEPACAM2: hepatocyte cellular adhesion molecule; ICAM2: intercellular adhesion molecule; IGF: insulin-like growth factor; ITGA4: integrin A4; JMJD6: jumonji domain containing 6; MET: hepatocyte growth factor; NRP1: neuropilin 1; PCDHGC3: protocadherin gamma subfamily C3; RIN: RNA integrity number; TBS: Tris-buffered saline; TGFB: tTransforming growth factor-beta; WHO: World Health Organization.

## Competing interests

The authors declare that they have no competing interests.

## Authors' contributions

All authors read and approved the manuscript. RK conducted the experimental work including microarray hybridization. RK and ADG designed the study, performed the histological analysis and the follow-up of the patients. DL, MH and RK accomplished the data analysis and prepared the manuscript.

## Pre-publication history

The pre-publication history for this paper can be accessed here:

http://www.biomedcentral.com/1471-2407/10/618/prepub

## Supplementary Material

Additional file 1 Sequences of primers used for qPCR Click here for file

Additional file 2 excel data sheet, gene list of differentially expressed genes in metastatic CMT, selection criteria included fold-change >2 or <-2 and q < 0.001 Click here for file
