# Supplementary material for: Metastatic canine mammary carcinomas can be identified by a gene expression profile that partly overlaps with human breast cancer profiles
Source: BMC Cancer. 2010 Nov 9;10:618. doi: 10.1186/1471-2407-10-618 (PMC2994823; doi:10.1186/1471-2407-10-618)
Supplement: Additional file 1 — Sequences of primers used for qPCR [file 1471-2407-10-618-S1.DOC]

## Supplemenatl 1 - Sequences of primers used for qPCR

| Gene | Primer sequence (5` - 3`) | Amplicon size | Accession no. |
| --- | --- | --- | --- |
| ATP5B | fw-GCACGGAAAATACAGCGTTT rev-TTGCCACAGCTTCTTCAATG | 186 bp | NM_001686 |
| AURKA | fw-TTGTCCTGCCTCTAGGTCAC  rev-AAAATCTTCCAAAGCCCATT | 115 bp | XM_848304.1 |
| ALOX12 | fw-GCTGCACCCTGTCTTCAA  TGCTCACCACCTTATCGAAT | 117 bp | ENSCAFT00000025298 |
| BMP-6 | fw-TTTTCCTGGTGAATGTGGTT rev-ATCCGTCCTTTCTCTGTTCC | 104 bp | XM_535880.2 |
| ERBB-4 | fw-CCTTGTCACTGGTATTCATGG  rev-GAAGTCGGTCATGTTTGGAG | 138 bp | XM_545629.3 |
| HEPACAM2 | fw-GCTCCAGTGACCAAAGAAGA  rev-CTCGAAGTCCATAAGGTCCA | 118 bp | ENSCAFT00000003183) |
| HPRT | fw-TGCTCGAGATGTGATGAAGG rev-TCCCCTGTTGACTGGTCATT | 191 bp | NM_000194 |
| IGFR2 | fw-ATTACAGGCACCAGGATGAA  rev-CTCGTAGCTCTTCCCATTGA | 122 bp | ENSCAFT00000001105 |
| RAD51 | fw- GGAGAAGGAAAGGCCATGTA rev- GGGTCTGGTGGTCTGTGTT | 148 bp | NM_001003043 |
| RP32 | fw- ATGCCCAACATTGGTTATGG rev-CTCTTTCCACGATGGCTTTG | 180 bp | XM_540107 |
| TGFBR-3 | fw-AGTGGTCAAAAATCTCATCCT rev-TCCAAAGCCAATACTGTTAG | 117 bp | XM_547284 |
